# Effect of Cleaning Protocol on Bond Strength between Resin Composite Cement and Three Different CAD/CAM Materials

**DOI:** 10.3390/ma13184150

**Published:** 2020-09-18

**Authors:** Nina Lümkemann, Lisa Marie Schönhoff, Ramona Buser, Bogna Stawarczyk

**Affiliations:** Department of Prosthetic Dentistry, Dental School, University Hospital, LMU Munich, 80336 Munich, Germany; nina.luemkemann@med.uni-muenchen.de (N.L.); lisa.schoenhoff@med.uni-muenchen.de (L.M.S.); ramona.buser@med.uni-muenchen.de (R.B.)

**Keywords:** cleaning protocol, bond strength, CAD/CAM materials, zirconia, lithium-disilicate, resin composite

## Abstract

The present investigation tested the effect of the cleaning method on the tensile bond strength (TBS) between one resin composite cement (RCC) and three different computer aided design/computer aided manufacturing (CAD/CAM) materials, namely zirconia, lithium disilicate ceramic and resin composite. Ninety specimens were prepared from each CAD/CAM material (N = 270). The specimens were pre-treated respectively, divided into five subgroups and subjected to five different cleaning protocols, namely i. 37% phosphoric acid, ii. ethanol, iii. phosphoric acid + ethanol, iv. cleaning paste, v. distilled water. After cleaning, the specimens were either conditioned using a universal primer or a universal adhesive and bonded using a dual-curing RCC. After thermo-cycling (20,000x at 5 °C/55 °C), TBS and fracture patterns were evaluated. The data was analyzed using 1- and 2-way Analysis of Variance (ANOVA) with post-hoc Scheffé and partial eta-squared (*ƞ_P_*²), Kruskal–Wallis, Mann–Whitney U and Chi^2^ tests (*p* < 0.05). The CAD/CAM material showed an impact on the BS while the cleaning protocol did not affect the results. Zirconia obtained the highest BS, followed by lithium-disilicate-ceramic. Resin composite resulted in the overall lowest BS. For most fracture patterns, the cohesive type occurred. All tested cleaning protocols resulted in same BS values within one CAD/CAM material indicating that the impact of the cleaning method for the restorative material seems to play a subordinate role in obtaining durable bond strength to resin composite cement. Further, it indicates that the recommended bonding protocols are well adjusted to the respective materials and might be able to compensate the impact of not accurately performed cleaning protocols.

## 1. Introduction

In modern dentistry, clinicians have a choice of many different restoration materials depending on the indication. Tooth-colored materials have become increasingly popular due to their esthetic properties. The group of esthetic materials mainly includes ceramics and polymers. Due to the ever-progressing computer aided design/computer aided manufacturing (CAD/CAM) technologies, as well as the innovative material development in terms of esthetics and mechanical properties, these materials are increasingly used for monolithic restorations [1]. The fabrication of a monolithic restoration is quick and easier compared to a veneered fixed dental prosthesis (FDP). These restorations are often luted using resin composite cements (RCC). However, this cementation type is more complex than cementing with conventional cements, e.g., glass ionomer cement. There are two bonding mechanisms when luting with RCC, namely the mechanical and chemical bonds. The mechanical bond is achieved by enlarging the surface via surface roughening in different ways. For glass-ceramics (leucite or lithium silicate ceramics), etching with hydrofluoric acid is preferred [2,3]; for oxide ceramics (zirconium oxide) or polymers, the surface is air-particle abraded [4,5,6]. After this mechanical pretreatment, the surface must be cleaned in order to obtain a durable bond strength [7,8]. For ceramics it was concluded that the surface cleaning methods should be applied according to different ceramic properties [9].

To clean the inner surface of air-particle abraded or acid-etched restorations, it is generally recommended to remove dust and any residuals of the air-particle abrasion or the acid agent [10,11,12]. Clinicians conventionally employ an oil-free air stream or air/water spray for the removal of loose particles, as recommended by some manufacturers [13,14]. Another method conventionally used in prosthetic clinics and research laboratories is the use of ultrasonic cleaning, which in one study has shown to significantly compromise the shear bond strength between zirconia and resin cement [15]. In a previous investigation the cleaning methods had little or no effect on the long-term resin bonding to zirconia when applying oil-free air or ultrasonic cleaning in alcohol as cleaning methods [16].

Universal cleaning agents have only been available for a short time now. According to the manufacturers, these have been developed for cleaning of various restoration surfaces after the pretreatment (etching, air-particle abrasion) in order to prevent incorrect cleaning. There are study results concluding that cleaning agents can improve the adverse effect of saliva contamination on zirconia but also indicate that the effect varies depending on the product [17]. After etching of glass ceramics using hydrofluoric acid, the surface is often cleaned with phosphoric acid. This method has been tried and tested and leads to good bond strength values [18]. In contrast to these outcomes, there are theories that cleaning zirconia restorations with phosphoric acid can negatively affect the bond strength to resin composite cements [19]. Phosphoric acid seems to be much more effective than cleaning just with alcohol [20], but a decrease of the bond strength is observed after artificial aging if phosphoric acid cleaning was used. This effect can be prevented by a subsequent cleaning step with alcohol [21,22,23]. The cleaning of zirconia surfaces with only phosphoric acid cannot be recommended if adhesive luting is desired. Whenever the surface is not cleaned sufficiently with alcohol following phosphoric acid treatment, the phosphate groups in the phosphoric acid might occupy oxide binding sites. These binding sites are usually available for the phosphate monomers contained in the self-etch resin composite cement or the adhesive system to form a chemical bond. 

Today’s polymer-based CAD/CAM materials are known to be challenging in regard of achieving sufficient bonding strength due to the high degree of conversion and the specific microstructure attained by the industrial production of resin composite materials [24]. Similar to zirconia ceramic, air-particle abrasion followed by the application of a certain adhesive (e.g., universal adhesive) is also recommended to achieve a sufficient bond strength to resin composite materials [25].

Irrespective of the restoration material, the effect of the cleaning method on the bonding durability after air-particle abrasion or etching is not well known. Some previous literature tested the effectiveness of different cleaning methods on contaminated zirconia [9,16,17,22,23,26,27,28,29,30], lithium disilicate ceramic [9,31,32,33] or a resin composite material [34]. However, the presented outcomes were controversial and mainly focused on the effectiveness of the cleaning method. 

Therefore, the aim of this study was to evaluate the effect of different cleaning protocols on the tensile bond strength (TBS) after artificial aging between resin composite cement and three different CAD/CAM materials, namely zirconia, lithium disilicate ceramic and resin composite. Zirconia and resin composite were air-particle abraded using alumina powder and lithium disilicate ceramic was etched using hydrofluoric acid. The five tested cleaning protocols included: (i) 37% phosphoric acid, (ii) ethanol, (iii) phosphoric acid + ethanol, (iv) universal cleaning paste, and (v) distilled water. The following null hypotheses were tested: (1) the cleaning protocol has no effect on the tensile bond strength between the restorative material and resin composite cement; (2) the cleaning protocol has no effect on the fracture type between the restorative material and the resin composite cement.

## 2. Materials and Methods 

**Figure 1:**
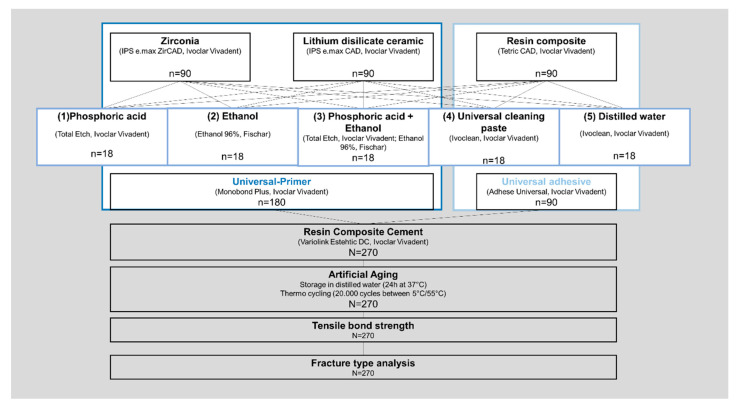
Study design: impact of cleaning protocol on the tensile bond strength between a resin composite cement and three different computer aided design/computer aided manufacturing (CAD/CAM) materials.

**Figure 2:**
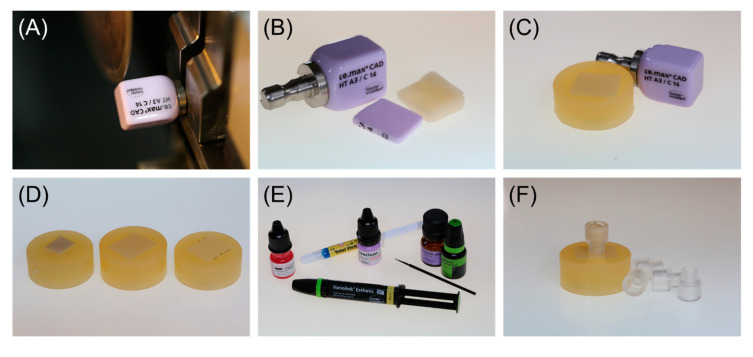
Step by step procedure of specimen preparation with (**A**) cutting of plate-shaped specimens with a cross section of 12.4 × 14.5 mm (lithium disilicate ceramic) and a thickness of 1.5 mm from CAD/CAM blocks using an automated cutting machine; (**B**) plate-shaped lithium disilicate specimens before and after crystallization; (**C**) embedded lithium disilicate ceramic specimen after polishing; (**D**) overview of all tested CAD/CAM specimens after application of cleaning protocols; (**E**) materials used for surface pretreatment and bonding with resin composite cement; and (**F**) acrylic cylinder bonded to specimen surface using resin composite cement.

A total number of 90 specimens was prepared from each material (Figure 1). All materials used are listed in Table 1. For zirconia (IPS e.max ZirCAD LT, Ivoclar Vivadent; Schaan, Liechtenstein), square-shaped blocks with a cross section of 7 × 7 mm and a thickness of at least 5 mm were cut from a pre-sintered CAD/CAM blank using a diamond wheel. Successively, the zirconia specimens were sintered according to the manufacturer’s instructions (Table 2; LHT 02/16, Nabertherm; Lilienthal, Germany). For lithium disilicate ceramic (IPS e.max CAD HT, Ivoclar Vivadent; Schaan, Liechtenstein) and resin composite (Tetric CAD, Ivoclar Vivadent; Schaan, Liechtenstein), plate-shaped specimens with a cross section of 12.4 × 14.5 mm (lithium disilicate ceramic, Figure 2A) or 11.8 × 13.8 mm (resin composite) and a thickness of 1.5 mm were cut from CAD/CAM blocks using an automated cutting machine (Secotom-50, Struers; Copenhagen, Denmark) equipped with a diamond-coated saw blade (M1D13, Struers; Copenhagen, Denmark). The cutting parameters were set at a feed speed of 0.05 mm/s and circulation speed of 2500 min^−1^. After cutting, the lithium disilicate specimens were crystallized (Figure 2B) according to the processing instructions provided by the manufacturer (Table 2; Programat EP5000, Ivoclar Vivadent; Schaan, Liechtenstein).

All specimens were singly embedded in acrylic resin (Figure 2D, ScanDiQuick A/B, ScanDia; Hagen, Germany) and polished according to a standardized polishing protocol using a series of silicone carbide papers up to P4000 under permanent water cooling (Tegramin-20, Struers; Copenhagen, Denmark). Afterwards, the specimens were ultrasonically cleaned with distilled water for 5 min (Transitor/Ultrasonic T-14, L&R; Kearny, NJ, USA).

Zirconia (n = 90) and resin composite specimens (n = 90) were air-particle abraded with alumina powder (50 µm) at a pressure of 0.1 MPa for 10 s (basic quattro IS, Renfert; Hilzingen, Germany). The surface of the lithium disilicate specimens (n = 90) was etched with 5% hydrofluoric acid (IPS Ceramic Etching Gel, Ivoclar Vivadent; Schaan, Liechtenstein) for 20 s and rinsed with distilled water. All specimens were ultrasonically cleaned with distilled water for 5 min after surface pre-treatment and allocated into the defined groups of cleaning protocols (Figure 1).

The cleaning was performed as follows:

1. Phosphoric acid

The 37% phosphoric acid (Total Etch, Ivoclar Vivadent; Schaan, Liechtenstein) was applied via drain tube onto the specimen surface and left for 15 s. Afterwards, the phosphoric acid was thoroughly rinsed with distilled water and the specimen surface was dried with oil-free air.

2. Ethanol

The specimen surface was immersed in ethanol (Ethanol 96%, V/V Otto Fischar; Saarbrücken, Germany) for 2 min and dried with oil-free air.

3. Phosphoric acid + ethanol

The 37% phosphoric acid (Total Etch, Ivoclar Vivadent; Schaan, Liechtenstein) was applied via drain tube onto the specimen surface, left for 15 s and thoroughly rinsed with distilled water. Afterwards, the specimen surface was immersed in ethanol (Ethanol 96%, V/V Otto Fischar; Saarbrücken, Germany) for 2 min and dried with oil-free air.

4. Universal cleaning paste 

The universal cleaning paste (Ivoclean, Ivoclar Vivadent; Schaan, Liechtenstein) was applied onto the specimen surface with a brush, left for 20 s and thoroughly cleaned with distilled water according to the instructions provided by the manufacturer. Afterwards, the specimen surface was dried with oil-free air.

5. Distilled water

No further cleaning procedure was applied after the specimens were ultrasonically cleaned for 5 min in distilled water after surface pre-treatment.

After performing the different cleaning protocols, the specimen surfaces were conditioned respectively (Figure 1, Table 1). For zirconia and lithium disilicate ceramic a universal primer (Monobond Plus, Ivoclar Vivadent; Schaan, Liechtenstein) was applied onto the specimen surface with a brush (Figure 2E), allowed to react for 60 s and gently dispersed with oil-free air. The resin composite specimens were conditioned with a universal adhesive (Adhese Universal, Ivoclar Vivadent; Schaan, Liechtenstein) that was evenly applied, allowed to react for 20 s, gently dispersed and light-cured for 10 s (Elipar S10, 3M; Saint Paul, MN, USA).

A transparent acrylic cylinder with an inner diameter of 2.9 mm was positioned in the center of the conditioned specimen surface, filled with resin composite cement (Variolink Esthetic DC, Ivoclar Vivadent; Schaan, Liechtenstein) and light-cured for 20 s (Elipar S10, 3M; Saint Paul, MN, USA) (Figure 2F). Excess resin composite cement was gently removed with a scalpel.

For artificial aging, the specimens were stored in distilled water in an incubator at 37 °C for 24 h (Hera cell 150, Kulzer; Hanau, Germany). After 24 h of water storage, all specimens were artificially aged in a thermo cycler for 20,000 cycles that were split between two water baths with distilled water (dwell time 30 s) at a temperature of 5 °C and 55 °C (THE-1100, SD Mechatronik; Feldkirchen, Germany). After 20,000 cycles of thermo-cycling, all specimens were dried in ambient air for 2 h before being subjected to TBS measurements.

The TBS was measured in a universal testing machine (RetroLine, Zwick/Roell; Ulm, Germany). For this, specimens were singly placed in a testing device without tension and a perpendicular load was applied with a crosshead speed of 5 mm/min until fracture of the specimen. The maximal force was recorded (testXpert, Zwick/Roell; Ulm, Germany) and the tensile bond strength calculated according to equation 1:

(1) σ=FmaxA

with *σ* (tensile bond strength in N/mm²), *F_max_* (load at fracture of bonding interface in N) and *A* (bonding area/interface in mm²).

All fracture patterns were analyzed under a light microscope at a magnification of 20× (Keyence VHX-S600E, Keyence; Ōsaka, Japan). The fracture type was defined according to following definition:

(A) *adhesive:* no residuals of resin composite cement on specimen surface

(B) *cohesive:* adhering residuals of resin composite cement on specimen surface

(C) *mixed:* adhering residuals of resin composite cement on specimen surface combined with fractures in specimen substrate

The measured data were analyzed using descriptive statistics. The normality of data distribution was tested using the Kolmogorov–Smirnov test, 1- and 2-way ANOVA with post-hoc Scheffé and partial eta-squared (*ƞ_P_*²). The Kruskal–Wallis and Mann–Whitney U tests were computed to determine the significant differences among the tested groups. Partial eta-squared was used to determine the effects of independent variables and interactions on the result of TBS. The relative frequencies of failure types together with the corresponding 95% confidence interval (CI) were provided according to the Ciba Geigy tables. The chi^2^-test was used to detect the differences in frequencies of failure types between the groups. The statistical tests were performed with a statistical software (IBM SPSS Statistics. v23.0, IBM Corp; Armonk, NY, USA).

## 3. Results

**Figure 3:**
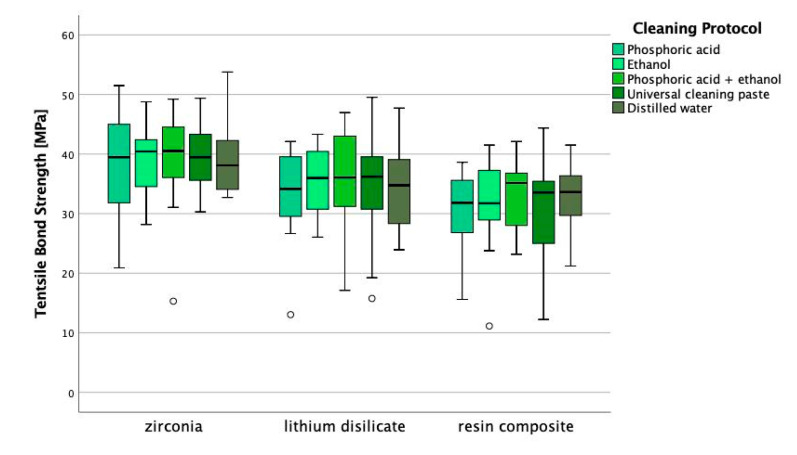
Tensile bond strength in MPa depending on the material and the cleaning protocol.

The Kolmogorov–Smirnov test showed deviations from the normal distribution for 6.67% of all tested groups. Groups that deviated from the normal distribution are indicated in Table 3. The type of material showed an impact on the tensile bond strength (*p* < 0.001; *ƞ_P_²* = 0.156) while the cleaning protocol did not affect the results (*p* > 0.766). Zirconia obtained the highest tensile bond strength, followed by lithium disilicate ceramic (*p* < 0.001). The CAD/CAM composite resulted in the overall lowest bond strength results (*p* < 0.003). Within the different CAD/CAM materials, no statistic differences in tensile bond strength were found in dependence of the cleaning protocol (*p* > 0.824; Figure 3), though an increased variance can be observed within lithium disilicate ceramic for cleaning with ethanol, universal cleaning paste, and distilled water, when compared with phosphoric acid or phosphoric acid + ethanol. Table 3 presents the descriptive statistics of the results of tensile bond strength according to the tested CAD/CAM materials and the applied cleaning protocol. The descriptive statistics include means, standard deviations, 95% confidence intervals as well as minimum, median and maximum (Table 3).

**Figure 4:**
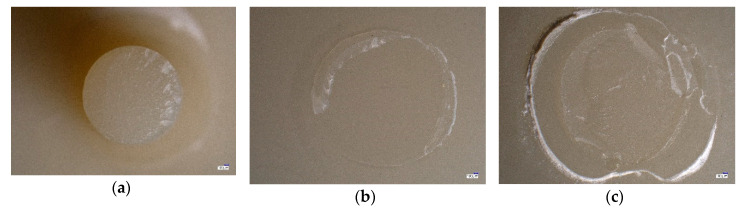
Overview of occurred fracture types according to the definition of (**a**) adhesive, (**b**) cohesive and (**c**) mixed.

Table 4 presents the results of the relative frequency of fracture types in % with the 95% confidence intervals according to the tested CAD/CAM material and the applied cleaning protocol. The characteristic fracture types defined in Table 4 are depicted in Figure 4. In most fractures, the cohesive fracture type occurred (Table 4). Adhesive fracture types occurred in some lithium disilicate and composite groups, mixed fracture types only occurred in two resin composite groups (Figure 4).

## 4. Discussions

In clinical practice, the cementation of a permanent restoration is a critical factor affecting the long-term success of the restoration and thus the patient’s satisfaction as well as his trust into clinical treatments. With the introduction of CAD/CAM technology the range of available permanent restorative materials rapidly grew, so that the clinician together with the cooperating dental laboratory can now choose from a wide range of restorative materials in order to serve the patient’s needs best. This increases the complexity of the correct processing of a restoration in the clinical practice, especially concerning the cementation/bonding procedure that varies for each material type. The cementation/bonding process usually includes several steps on both sides, the tooth as well as the restoration, before the resin composite cement is applied: preparation followed by a mechanical and/or chemical pretreatment, appropriate cleaning followed by the application of a primer/adhesive. 

The present in-vitro investigation clearly followed the recommended bonding protocols respective to the restorative materials and focused on the impact of different cleaning protocols on the bond strength between resin composite cement and three different CAD/CAM materials (zirconia, lithium disilicate ceramic and resin composite). Based on the overall results of the present investigation, both tested null hypotheses could be accepted. With respect to the different tested restorative materials, the cleaning protocol neither affected the bond strength nor the fracture types between the respective material and resin composite cement. This outcome partly coincides with the results found in previous investigations, in which the impact of the cleaning protocol is controversially discussed [9,23,27,28,29,30,32,33]. In compliance, most investigations summarized that the contamination with saliva negatively affects the bond strength between a restorative material and resin composite cement [9,27,29].

Within the present investigation, the substrates were not contaminated with saliva or blood to solely test the impact of the cleaning protocol. At first, this might be noticed as limitation of the present investigation, since the contamination with saliva and/or blood often happens during the try in of the restoration in the clinical practice and therefore is considered in several previous investigations when testing the impact of different cleaning protocols on the bond strength between dental materials and resin composite cement [29,30]. However, in comparison to previous studies, the aim of the present investigation was not to evaluate the efficacy of the tested cleaning protocols after any kind of contamination but to analyze the impact of the cleaning protocol on the bond strength between resin composite cement and the respective restorative material when following the accurate bonding protocol. To the best of the authors knowledge, there is no comparable investigation in literature. Some previous studies included a non-contaminated, non-cleaned group as positive control group in their study designs [29,30]. Here, some cleaning protocols resulted in comparable bond strength values to the positive control group after surface contamination and were therefore evaluated as being most effective.

For zirconia it was assumed that cleaning with phosphoric acid might negatively affect the bond strength to resin composite cement [19]. It was suspected that the phosphate groups in the phosphoric acid might occupy oxide binding sites which should usually be available for the phosphate monomers contained in the self-etch resin composite cement or the adhesive system to form a chemical bond. To avoid this effect, subsequent cleaning of the zirconia surface with alcohol was recommended [21,22,23]. The present results neither prove a negative effect of cleaning of zirconia using phosphoric acid nor prove any benefit of sole or subsequent cleaning with alcohol. On the one hand, previous results found that the saliva contamination of zirconia could only be eliminated by additional cleaning with either air-particle abrasion or the application of an “AD Gel”, while water-rinsing or the application of a cleaning paste (Ivoclean; Ivoclar Vivadent) did not result in a durable resin bonding to zirconia [23]. On the other hand, other researchers found no differences between different tested cleaning protocols for resin bonding to zirconia which is consistent to the present results [27]. Here, the tested cleaning protocols include water-rinsing, the usage of isopropanol or air-particle abrasion as well as the application of Ivoclean or Monobond Plus, which is comparable to the methods used in the present investigation. Thus, the present outcomes for zirconia are partly confirmed. Further two studies confirmed the positive effect of the tested cleaning paste on the bond strength and the ability to remove saliva and blood contamination [29,30]. Alternatively, the use of nonthermal atmospheric plasma [29] or airborne-particle abrasion [30] was also found to be effective in removing saliva and blood contamination on zirconia.

For lithium disilicate ceramic or glass ceramics in general, the cleaning with phosphoric acid is a well-tested method that shows positive results on bond strength values [18]. The common approach to bond lithium disilicate restorations includes etching with 5–9.5% hydrofluoric acid for 20–30 s in order to create a micro retentive increased surface with a high number of functional silane groups. The silane groups are important for the chemical interaction with the functional groups of the silane primer. The silane primer should be applied on the etched ceramic surface after thorough cleaning with phosphoric acid. The obtained results showed that the use of alcohol or water is as effective as the use of phosphoric acid. This confirms previous findings where no impact of different cleaning methods was found for lithium disilicate ceramic as well as for leucite-reinforced glass ceramics [9]. Though no statistic differences could be found, it has to be noted that in this investigation the variance in TBS was lower for cleaning protocols including phosphoric acid within lithium disilicate ceramic (Figure 3). This might indicate that utilizing phosphoric acid would be more reliable as cleaning procedure. These findings could possibly be confirmed in a comparable study, where after thermal aging variance also seems to be slightly reduced for groups cleaned with phosphoric acid compared to the other groups, but to verify statistical significance of this tendency a Weibull analysis would have had to be performed. This study tested several cleaning methods including “K etchant Gel”, Ivoclean, “AD Gel” as well as the application of a silane coupling agent before immersion in saliva and proves that all methods result in a durable bond strength to resin composite cement. Here, water rinsing was the only method showing a negative impact on the bond strength before and after aging [33]. Since sole rinsing with water did not result in a durable bond strength between glass ceramics and resin composite cements, it is recommended to first rinse the saliva contaminated surface with water and to subsequently treat the surface with a fresh layer of silane primer [32]. This is consistent with the approach used in this investigation. 

One of the best-known universal primers is Monobond Plus (Ivoclar Vivadent), which has also been used in the present investigation to pretreat zirconia and lithium disilicate ceramic. The present results showed that none of the tested cleaning protocols, irrespective of zirconia or lithium disilicate ceramic, revealed superior bond strength values, which might indicate the high effectiveness and efficiency of the used silane primer Monobond Plus. This observation was also confirmed by the fracture type analysis. The predominant occurrence of the cohesive fracture type (Figure 4) indicated that the bond strength in the interface between the restorative material and the resin composite cement was higher than the inherent strength of the resin composite cement itself.

Compared to zirconia, the tested resin composite was pretreated differently after air-particle abrasion compared to zirconia by using a universal adhesive, namely Adhese Universal (Ivoclar Vivadent). The approach used here, is in accordance to the recommendations of a meta-analysis suggesting that for resin composite materials alumina air abrasion followed by a silane coupling agent or universal adhesive could be considered the best strategy to yield optimal bond strength results after artificial aging [25]. Universal adhesives are one-bottle systems that include different reactive components like dimethacrylate, silane, phosphate- and sulfuric monomers as well as methyl methacrylate and can therefore be used for several restorative materials. The result of the present investigation, that also none of the tested cleaning protocols negatively affected the bond strength of the resin composite material to the resin composite cement, proved that the used bonding protocol as well as the applied products generate a reliable bond strength. Herein again, the analysis of the fracture types confirms the high bond strength of the interface between restorative material and resin composite cement. The fact that also mixed fracture types occurred within the group of resin composite, indicates that the bond strength on the interface is not only higher than the inherent strength of the resin composite cement but also exceeds the strength of the restorative material itself. The reason, that the resin composite cement resulted in the lowest overall bond strength values compared to zirconia and lithium disilicate ceramic might be based on the fact that the adhesive bonding of resin composites to resin composite cement is challenging due to the high degree of conversion and the specific microstructure achieved by the industrial production of resin composites at high manufacturing temperatures and pressures [24]. 

## 5. Conclusions

The outcomes of the present investigation proved that the recommended bonding protocols for adhesive cementation are very well adjusted to the respective materials. The investigated bonding materials might be able to compensate the impact of not correctly performed cleaning protocols. Thus, the compliance with the correct cleaning protocol seems to be negligible as long as the clinician is familiar with the bonding protocol for the chosen material, meaning that the pretreatment steps are precisely followed and the bonding materials are processed according to the instructions provided by the manufacturer. If this is the case, the present outcome would be highly beneficial, simplifying the highly complex process of adhesive cementation of restorations for the clinician. In conclusion, the impact of the chosen cleaning method seems to play a subordinate role in obtaining a durable bond strength to resin composite cement as long as the clinician complies with the bonding protocol of the respective restorative material and handles the bonding materials correctly.

## Figures and Tables

**Table 1:** List of the materials used according the product name, manufacturer, chemical composition and Lot No.

Material	Product Name	Manufacturer	Chemical Composition	Lot No.
Lithium disilicate glass-ceramic	IPS e.max CAD HT	Ivoclar Vivadent; Schaan, Liechtenstein	Lithium disilicate glass-ceramic (LS_2_) Silicate-based glass-ceramic	X25830
Zirconia	IPS e.max ZirCAD LT	Ivoclar Vivadent; Schaan, Liechtenstein	ZrO_2_ 88.0–95.5 wt.% Y_2_O_3_ > 4.5 ≤ 6.0 wt.% HfO_2_ ≤ 5.0 wt.% Al_2_O_3_ ≤ 1.0 wt.% Other oxides ≤1 wt.%	X17186
Resin composite	Tetric CAD	Ivoclar Vivadent; Schaan, Liechtenstein	Cross-linked dimethacrylate inorganic filler particles	X12627
Hydrofluoric acid	IPS Ceramic Etching Gel	Ivoclar Vivadent; Schaan, Liechtenstein	<5 wt.% hydrofluoric acid	W14921
Phosphoric acid	Total Etch	Ivoclar Vivadent; Schaan, Liechtenstein	37 wt.% phosphoric acid in water, thickening agent, color pigments	X51245
Cleaning paste	Ivoclean	Ivoclar Vivadent; Schaan, Liechtenstein	Dispersion of metal oxide particles in water	X47350
Universal primer	Monobond Plus	Ivoclar Vivadent; Schaan, Liechtenstein	Alcohol solution of silane methacrylate, phosphoric acid methacrylate and sulphide methacrylate	X51129
Universal adhesive	Adhese Universal	Ivoclar Vivadent; Schaan, Liechtenstein	methacrylates, ethanol, water, highly dispersed silicon dioxide, initiators and stabilizers	X41517
Resin composite cement	Variolink Esthetic DC	Ivoclar Vivadent; Schaan, Liechtenstein	Monomer matrix: urethane dimethacrylate, and further methacrylate monomers; inorganic fillers: ytterbium trifluoride and spheroid mixed oxide; initiators: stabilizers, pigments	X41008

**Table 2:** Sintering parameters used for IPS e.max ZirCAD (LHT 02/16, Nabertherm; Lilienthal, Germany) and IPS e.max CAD (Programat EP5000, Ivoclar Vivadent; Schaan, Liechtenstein) according to the manufacturer’s instructions.

Material	Start Temperature (°C)	End Temperature (°C)	Heating/Cooling Rate (°C/min)	Holding Time (min)
IPS e.max Zir CAD	20	900	10	30
	900	1500	3.3	120
	1500	900	10	---
	900	300	8.3	---
IPS e.max CAD	550	550	0	6
	550	820	90	1
	820	840	30	7
	840	700	---	---

**Table 3:** Descriptive statistics of tensile bond strength in MPa with mean **±** standard deviation (SD), 95% confidence interval (CI), minimum, median, maximum according to the CAD/CAM materials and the applied cleaning protocol.

Material	Cleaning Protocol	Tensile Bond Strength (MPa)		
		Mean ± SD	95% CI	Min/Median/Max
IPS e.max Zir CAD	Total Etch	37.5 ± 9.2	32.8;42.1	20.9/39.5/51.5
	Total Etch + Ethanol	39.1 ± 7.6	35.1;42.9	15.3/40.5/49.2
	Ethanol	38.9 ± 5.6 *	36.0;41.7	28.2/40.4/48.8
	Ivoclean	39.6 ± 4.7	37.1;42.0	30.3/39.5/49.4
	Distilled Water	39.2 ± 5.6	36.3;42.1	32.7/38.1/53.8
IPS e.max CAD	Total Etch	33.5 ± 7.1	29.9;37.1	13.0/34.2/42.1
	Total Etch + Ethanol	35.8 ± 7.6	31.9;39.6	17.1/36.1/47.0
	Ethanol	35.4 ± 5.4	32.5;38.1	26.1/36.0/43.3
	Ivoclean	34.0 ± 9.0	29.3;38.4	15.8/36.2/49.5
	Distilled Water	35.2 ± 7.4	31.4;39.0	23.9/34.8/47.7
Tetric CAD	Total Etch	31.1 ± 6.0	27.9;34.1	15.6/31.8/38.6
	Total Etch + Ethanol	33.3 ± 5.7	30.3;36.2	23.2/35.1/42.1
	Ethanol	31.7 ± 7.3	28.0;35.4	11.1/31.7/41.5
	Ivoclean	30.8 ± 7.7	26.8;34.7	12.2/33.6/44.4
	Distilled Water	32.5 ± 5.1	29.8;35.1	21.2/33.6/41.5

* indicates deviation from normal distribution.

**Table 4:** Relative frequency of fracture types in % with 95% CI.

Material	Cleaning Protocol	Fracture Type		
		Adhesive	Cohesive	Mixed
IPS e.max Zir CAD	Total Etch	0 (0;19)	100 (80;100)	0 (0;19)
	Total Etch + Ethanol	0 (0;19)	100 (80;100)	0 (0;19)
	Ethanol	0 (0;19)	100 (80;100)	0 (0;19)
	Ivoclean	0 (0;19)	100 (80;100)	0 (0;19)
	Distilled Water	0 (0;19)	100 (80;100)	0 (0;19)
IPS e.max CAD	Total Etch	11 (1;35)	89 (64;99)	0 (0;19)
	Total Etch + Ethanol	0 (0;19)	100 (80;100)	0 (0;19)
	Ethanol	0 (0;19)	100 (80;100)	0 (0;19)
	Ivoclean	6 (0;28)	94 (71;99)	0 (0;19)
	Distilled Water	0 (0;19)	100 (80;100)	0 (0;19)
Tetric CAD	Total Etch	11 (1;35)	89 (64;99)	0 (0;19)
	Total Etch + Ethanol	0 (0;19)	100 (80;100)	0 (0;19)
	Ethanol	0 (0;19)	94 (71;99)	6 (0;28)
	Ivoclean	0 (0;19)	100 (71;99)	6 (0;28)
	Distilled Water	0 (0;19)	100 (80;100)	0 0 (0;19)
